# Machine learning driven identification of optimal nanomaterials for efficient pararosaniline dye removal from water using a RFHGB hybrid model

**DOI:** 10.1039/d5ra09598k

**Published:** 2026-03-04

**Authors:** Ganesan Anandhi, M. Iyapparaja

**Affiliations:** a Department of Smart Computing, School of Computer Science Engineering and Information Systems, Vellore Institute of Technology Vellore 632014 Tamil Nadu India iyapparaja.m@vit.ac.in +919942532920

## Abstract

Water pollution by emerging contaminants requires advanced treatment technologies aside from conventional approaches due to the particular threat they pose to environmental and public health. Pararosaniline dye pollutant (PRS) is generally used in textile and biological staining applications, which may result in strong chemical stability, low biodegradability, and high toxicity, making its complete removal from wastewater so difficult. In this study, a ZnO–CuO nanocomposite and SrO photocatalysts were synthesized by experimental means and evaluated for photocatalytic degradation of PRS under controlled conditions. A dataset consisting of 81 experimental observations was computationally expanded to 5000 using synthetic data augmentation. Fifteen machine learning algorithms were trained to predict degradation efficiency, and the top five models were identified based on their performance metrics. Pairwise hybridization of the best five models produced ten hybrid combinations, out of which the Random Forest + HistGradient Boosting hybrid model (RFHGB-hybrid model) demonstrated the highest accuracy and lowest prediction error. The model also provided optimal degradation conditions and catalyst ranking, finding ZnO–CuO to be the best-performing photocatalyst.

## Abstract 2

An RFHGB machine learning model integrated with synthetic data augmentation accurately predicts photocatalytic degradation of pararosaniline. It also identifies ZnO–CuO as the most efficient catalyst.

## Graphical abstract

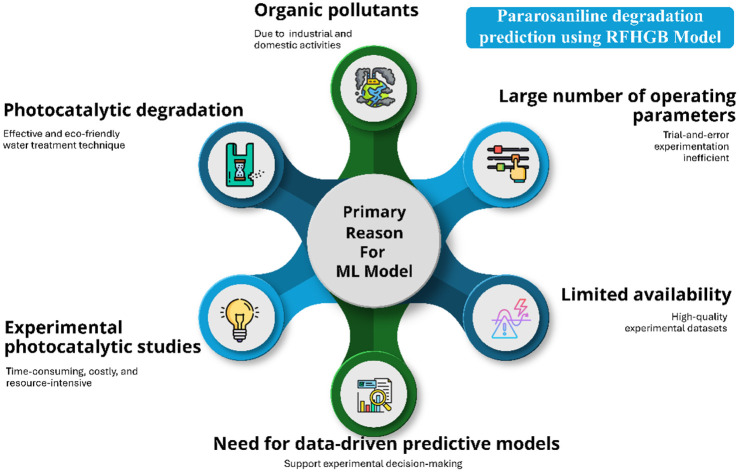

## Introduction

In this context, the water pollution caused by emerging contaminants has become one of the most persistent and critical challenges in modern environmental science.^1,2^ The rapid industrial expansion, pharmaceutical consumption, agricultural runoff, and urbanization have disproportionately increased the entry of complex organic pollutants into the aquatic systems.^3^ Further, the properties of structural stability, low biodegradability, and high persistence of such contaminants often reduces or nullifies their complete removal by conventional wastewater treatment technologies based on activated sludge processes, chemical precipitation, and membrane filtration.^4–6^ Therefore, in the last two decades, the efforts of researchers have focused on innovative treatment strategies capable of ensuring complete mineralization of hazardous pollutants, rather than their partial removal or phase transfer. In this context, the global environmental scientific community has underlined the urgent need for advanced oxidation processes and catalytic systems capable of efficient management of such persistent pollutants.^7,8^

Among the broad class of pollutants, the well-known dye PRS pollutant has drawn enormous scientific interest.^9^ Its wide application in pharmaceutical formulations and for treating bacterial infections in livestock farming and aquaculture is the reason for its excessive release into natural water bodies.^10,11^ The high chemical stability, resistance to biodegradation, and slow natural decomposition of the compound tend to keep it intact longer in soil and freshwater environments. Literature studies have documented its bioaccumulation within ecosystems, adversely affecting microbial communities, disrupting plant metabolic pathways, and creating a toxic response in aquatic organisms.^12–14^ Continuous exposure to PRS is also associated with the development of antimicrobial resistance – one of the major public health concerns worldwide.^15^ It hence has become important to develop such advanced technologies which allow the complete degradation of PRS into non-toxic intermediates for sustainable environmental protection. Recent machine learning research on dye adsorption by hydrochar and biochar-based systems offers a promising application domain for ML use for wastewater treatment purposes, while also drawing attention to some encountered obstacles regarding accessible information.

### PRS dye: toxicity and need for efficient degradation

PRS is a triphenylmethane dye whose applications are very extensive in the textile, biological staining agent, paper printing, and synthetic chemical industries. However, because of its molecular nature, it is considered a highly potent carcinogen and mutagen due to the continuous binding of the pararosaniline molecule with proteins.^16^ When discharged into wastewater, the pararosaniline exhibits extreme colour intensity, high photostability, and resistance to natural biodegradation pathways. Even low exposure concentrations have adverse impacts on aquatic organisms by disrupting enzyme function, impairing reproduction, and making changes in respiratory systems. Chronic exposure in humans results in skin irritation, respiratory distress, and haematological disorders, and has been strongly implicated with carcinogenic changes.

The persistence and harmful effects of pararosaniline stress the urgent need for advanced degradation techniques capable of complete mineralization of dye molecules instead of transferring them to another phase by adsorption or chemical conversion. Coagulation, flocculation, and activated carbon absorption remove the visible color but do not eliminate the toxic chemical structure responsible for mutagenicity. Therefore, photocatalysis has emerged as a fast and eco-friendly alternative degradation technique that degrades complex dye molecules *via* reactive oxygen species under the influence of light irradiation into harmless byproducts like carbon dioxide and water. Complete degradation requires optimization on catalyst material, dosage, pH conditions, light intensity, and exposure time.^17^

### Metal oxide nanocomposites for photocatalysis: focus on ZnO–CuO and SrO

Nanocomposites of metal oxides have, in recent times, emerged as highly efficient photocatalysts due to their optical properties, surface activity, and charge transport characteristics. Among commonly studied materials, ZnO and CuO have indeed demonstrated superior photocatalytic properties owing to better stability, a wide band-gap absorption range, and excellent electron mobility.^18–22^ While ZnO nanoparticles exhibit strong UV light absorption, CuO has a relatively narrower band gap for visible-light activation. Once combined as a ZnO–CuO nanocomposite, they show a heterojunction structure that extensively enhances the efficiency of charge separation, reduces electron–hole recombination, and thus increases the rate of radical formation. This synergistic interaction accelerates organic pollutant degradation, such as PRS and industrial dyes.^7,8,23^

SrO is another emerging photocatalyst that has drawn the attention of researchers in recent times due to its tunable optical response and excellent surface charge behavior. It has shown some promise toward wastewater treatment applications, simply because of its highly alkaline nature and creation of abundant reactive oxygen species upon irradiation. Surface defect engineering has been favorable toward SrO, unlike in the case of most conventional metal oxides; it, in fact, enhances the photocatalytic activity and stability of the material.^18^ A comparison of ZnO–CuO nanocomposites with SrO has provided insight into bandgap engineering, structural modification, and charge transfer behavior. These differences give important knowledge about choosing the most efficient catalyst for maximum PRS degradation efficiency.^24^ As the literature survey demonstrated that, SrO is less stable and immediately it forms Sr(OH)_2_ which results in less percentage of degradation and difficult to recover, hence the authors have chosen ZnO–CuO nanocomposite for better results.

### Role of machine learning in catalyst selection

Machine learning has already revolutionized scientific research by offering a way to assess and optimize complex systems with very high accuracy, something that in the past would have been achieved only through tiresome experimentation.^25^ In photocatalysis, the performance of the catalyst is controlled by several interacting parameters, such as catalyst dosage, dye concentration, pH, irradiation time, light intensity, surface area, and band-gap energy. The traditional experimental methodology relies on a trial-and-error search, which is rather time-consuming and expensive. It cannot screen the entire design space either. It is here that machine learning offers the new capability to analyze huge datasets, identify hidden relationships, and make reliable predictions of degradation efficiency across variable conditions. During the last years, ML evolved to become the major tool to guide photocatalyst selection and performance evaluation. Combining literature data with experimental data, machine learning models are able to classify and rank the best combinations of catalysts for maximum dye degradation. Synthetic data generation further expands this dataset to improve the stability and generalization of the models. Different predictive algorithms (15 nos) have shown very good accuracy in modeling nonlinear relationships inherent in photocatalytic reactions. Therefore, machine learning offers routes for accelerated material discovery and data-driven decision making for improved environmental treatment technologies.

The characterization results verify the catalyst synthesis, composition, and stability, proving the applicability of the experimental degradation data used for modeling. This information was not directly included.

### Objectives of the present study

The present study focuses on the development of a hybrid machine learning framework that can predict catalyst performance and determine the optimal material conditions for the photocatalytic degradation of PRS pollutant. Literature-derived datasets of SrO and experimentally generated ZnO–CuO nanocomposite data are collected and combined for this purpose. Experimental and synthetic datasets covering broad domains of operating variables would be extended to simulate those affecting photocatalytic efficiency in this research work. In this way, efficient model training is possible, and higher reliability in the prediction of catalyst performance will also be ensured. Another important objective is to investigate the performance of several machine learning algorithms and to find the best hybrid approach that guarantees high accuracy in prediction with low error metrics. The presented work will investigate ten base algorithms that have been selected to model hybrid frameworks using weighted averaging, stacking, and voting regressions. Eventually, a data-driven approach will be developed that should be able to predict the optimal reaction conditions, rank the catalysts, and minimize experimental effort. The current study also contributes to the advancement of computational tools within environmental engineering by practically integrating synthetic data, ensemble learning, and material optimization. Photocatalytic degradation experiments were carried out following a series of standardized laboratory conditions and the chosen catalyst dosages, initial PRS concentrations, and photocatalytic degradation times. The degradation efficiency of the photocatalytic reaction was recorded at a series of 10-minute intervals to track the reaction trend. The standardized degradation experiments and conditions pertain to discrete rather than a factorial series of conditions.

Due to the constraints in catalyst synthesis and the experimentation rate, extensive replicate experiments could not be carried out in this investigation. However, the experiments were done under similar operating conditions to provide reproducibility for the degradation profiles. This limitation is recognized, and the implications in statistical robustness are addressed accordingly.

## Materials & methods

### Data collection

The data required for this study were collected from experimental work and authenticated scientific literature. In the present study, two photocatalysts were considered experimentally, one namely ZnO–CuO nanocomposite (synthesized in lab and collected 18 nos of data) and another is SrO nanoparticles (taken from literature survey (IEEE data port – 63 nos of data)^26^ and the hyperlink of the dataport has been included in the SI document), which were tested for their efficiency in degrading PRS under controlled laboratory conditions. In all, 81 experimental observations were recorded by changing different reaction parameters, such as catalyst dosage, initial pollutant concentration, pH of the reaction medium, irradiation time, and type of catalyst. Degradation efficiency was quantitatively determined through UV-Vis spectrophotometry by observing the decrease in absorbance at characteristic wavelengths.

In parallel, reference-based data corresponding to SrO photocatalysis were collected from the published literature to supplement the comparative analysis. Experimental variables and measurement units were ensured to agree with the current dataset to allow consolidation in a uniform manner. The resultant combined dataset then constituted the baseline dataset, which was computationally expanded in this work for the purpose of machine learning model development and generalization performance. In this respect, the dual-catalyst dataset thus allowed for a proper comparative evaluation and prediction of catalyst efficiencies under a wide range of operating conditions.

### Data pre-processing

The preprocessing of the raw data was quite extensive in order to prepare it for machine learning implementation. It is common for raw data to include missing or inconsistent entries due to fluctuations in measurement or incomplete reporting. For these, treatments using imputation statistically guided by feature correlations and localized averaging were employed. Outliers were analyzed through interquartile and *z*-score methods so as not to distort model learning. Normalization through standard scaling has been used to maintain consistency in numerical behavior across features with various scales during model training.

Feature engineering was done in order to identify and retain only those parameters that influence degradation efficiency the most. Considering experimental evidence and statistical feature importance ranking, some critical variables were selected: initial dye concentration, catalyst dosage, pH of the solution, irradiation time, light intensity, band-gap energy, and specific surface area. This not only improved the interpretability of the model but reduced dimensional complexity for better computational efficiency. Thus, the structured dataset represented the basic input matrix for subsequent machine learning analysis. Prior to modeling, missing values were found and treated using mean imputation. Statistical thresholds were able to catch outliers, which then were removed. Since this is a pretty small dataset, one might expect that preprocessing decisions can significantly influence model stability and predictions.

### Machine learning framework

Since the experimental dataset had only 81 records, it was too small to train robust machine learning models that could capture complex nonlinear behavior and broad operational domains. In this respect, the dataset has been extended by computational data augmentation techniques up to 5000 synthetically generated points. Synthetic data generation relied on statistical bootstrapping in combination with SMOTE regression mechanisms in order to create new data points that corresponded to realistic reaction conditions according to the variation patterns identified in the original dataset. This approach allowed the simulation of experimental-like variability without the introduction of physically unrealistic values.

Synthetic data augmentation was used, strictly applying within experimentally determined parameter ranges, to stabilize machine learning training in a data-scarce regime. This approach does not add new mechanistic information. Additionally, it has the disadvantage that existing biases could be strengthened, especially considering the heterogeneous nature of the original dataset.

For validating the synthetic dataset, a comparison of distributions, kernel density estimation, and correlation heat-mapping were done to check consistency between the real and generated datasets. These statistical alignments showed that the extended dataset preserved the characteristics of the original data and the inter-feature relationships. This augmented 5000-point dataset provided a solid backbone for the training of machine learning models, enhancing reliability and generalization significantly in predictive modeling without further laboratory experiments.

### Base algorithm training (15 algorithms)

To predict the photocatalytic degradation efficiency, a broad machine learning framework was implemented using a set of 15 machine learning algorithms. Thus, the workflow that can be seen includes Random Forest (RaFo),^27,28^ Extra Trees (ExTr),^29^ Decision Tree (DeTr),^30^ AdaBoost (AdBo),^31,32^ Gradient Boosting (GrBo),^33,34^ HistGradient Boosting (HiBo),^35^ K-Nearest Neighbors (KNN),^25^ Support Vector Regression (SuVeRe),^36,37^ Linear SVR (LiSVR),^38^ Kernel Ridge (KeRi),^39^ Ridge Regression (RiRe),^40^ Lasso (La), ElasticNet (ElNe),^41^ XGBoost (XGBo),^42^ and LightGBM (LiGBM).^43^ Each algorithm was trained and evaluated with the expanded 5000-point dataset; cross-validation was applied to ensure a fair comparison of performance. The *R*^2^, RMSE, MAE, and explained variance scores were used to evaluate the performance of each model. Details of the above algorithms have been included in the SI document. As the intrinsic properties of the catalyst, band gap, and surface area remained the same for all the samples, these factors did not directly contribute to the regression. These factors have been included to give a physical interpretation.

### Selection of top 5 algorithms

Based on the comparative analysis, RaFo, ExTr, GrBo, HiBo, and XGBo were selected as the best five models showing the highest prediction performance. These models indeed yielded better accuracy and lower error rates due to their ability to capture nonlinear feature interactions and complex behaviors of variables within photocatalytic systems. Residual analysis also confirmed that these five models give the most stable predictions across the dataset.

## Hybrid model development (pairwise fusion of top models)

Further, to improve the predictive capability, all possible combinations of the top five algorithms have been subjected to pairwise hybridization. The hybrid models are developed using weighted averaging and stacked ensemble integration; a total of ten hybrid combinations have been produced. Each model benefited from the base complementary strengths in minimizing the error propagation and realizing better overall accuracy. On the basis of the performance comparison, it was determined that the hybrid combination DeTr + XGBo always outperformed others with the best *R*^2^ and the lowest RMSE and MAE for overall 5000 datasets, but as the laboratory condition prefers neutral pH (for pH = 7), the best fit hybrid algorithm was found to be RaFo + HiBo (RFHGB).

### Evaluation metrics and assessment

Performance evaluation was performed with the help of statistical metrics, including *R*^2^ score, RMSE, MAE, residual distribution plots, and learning curves. They quantitatively validated the predictive strength and generalization capability. The best hybrid model proved the superiority of machine learning-driven catalyst evaluation as a practical approach to environmental material selection and process optimization.

The photocatalytic degradation study was conducted for two catalysts, ZnO–CuO nanocomposite and SrO nanoparticles, under controlled laboratory conditions. The percent degradation of PRS pollutant was recorded at regular time intervals and revealed that the degradation continuously increased with increased irradiation time and at an optimized dose of the catalyst. ZnO–CuO nanocomposite showed highly improved photocatalytic activity due to efficient charge separation because of the heterojunction between ZnO and CuO phases. This structure allowed superior electron transfer passageways and reduced electron–hole recombination, increasing the efficiency of degradation even in the case of moderate light intensity. On the other hand, the photocatalytic activity shown by SrO was found to be moderate due to the larger band-gap and lesser capacity of absorbing visible light, although the alkaline nature of the surface of the catalyst supported the effective formation of radicals.

The photocatalytic mechanism of ZnO–CuO was assigned to the generation of reactive oxygen species, mainly hydroxyl radicals and superoxide ions, which degraded PRS into simpler chemical intermediates. The experimental results obtained in this work have demonstrated that catalyst composition and pH value, together with irradiation time, played an important role in controlling degradation kinetics. ZnO–CuO showed higher degradation rates and better mineralization capability than SrO under identical experimental conditions. These observations provided strong justification for using machine learning models to predict optimal catalyst performance and to identify the best operational range to maximize pollutant removal.

### Dataset expansion and synthetic data validation

Only 81 data points from each catalyst in the experimental data were not sufficient to effectively train more advanced machine learning models, especially for predictive simulations at wider parameter spaces. A computationally synthesized dataset with 5000 data points, using statistical bootstrapping based synthetic sampling, was therefore devised to supplement this deficiency, ensuring realistic physical and chemical bounds. With this enlarged data set, much greater variability over factors such as catalyst dosage, pollutant concentration, pH, light intensity, and irradiation time could be introduced, therefore leading to better learning of complex interactions.

Validation of the synthetic dataset was performed by comparing the statistical distribution and correlation behavior of real and augmented datasets. Kernel density estimation profiles indicated a close alignment of original and synthetic data, confirming that the distribution was preserved. Feature correlation heatmaps demonstrated similar interaction patterns across experimental and expanded datasets, eliminating concerns about artificial noise and bias. This confirmed the synthetic augmentation as a reliable method to enhance machine learning generalization for performance prediction.

### Performance of 15 base machine learning algorithms

A total of fifteen machine learning algorithms, including RaFo, ExTr, DeTr, AdBo, GrBo, HiBo, KNN, SuVeRe, LiSVR, KeRi, RiRe, La, ElNe, XGBo, and LiGBM, were used to predict photocatalytic degradation efficiency based on integrated experimental and synthetic datasets. Their performances were judged by the *R*^2^, RMSE, MAE, and accuracy of cross-validation (Table 1). As expected, tree-based ensemble algorithms and their Boosting variants performed better than other linear and kernel-based algorithms owing to their better capability in capturing more nonlinear behaviors and complex interactions of variables inherent in photocatalytic modeling.

**Table 1:** Photocatalytic degradation efficiency based on integrated experimental and synthetic datasets for base models

ML model	Train *R*^2^	Val *R*^2^	Test *R*^2^	Train MAPE	Val MAPE	Test MAPE
RaFo	0.999937	0.999731	0.999528	2.833166	3.929039	4.934916
ExTr	1	0.997469	0.997756	0.003434	5.547822	6.97576
DeTr	1	0.999555	0.998741	0.003411	4.565375	6.844859
AdBo	0.954616	0.953428	0.957139	63.96692	39.67413	45.30753
GrBo	0.997971	0.997866	0.99791	13.55444	6.901981	16.66117
HiBo	0.999899	0.999727	0.999674	3.760146	4.513053	5.635602
KNN	0.955415	0.937771	0.932871	33.49992	28.46203	43.48513
SuVeRe	0.94133	0.936424	0.936908	86.58894	61.15478	67.00289
LiSVR	0.927858	0.924039	0.927986	72.23623	44.40328	72.85915
RiRe	0.927164	0.923662	0.926278	79.61056	47.47469	77.17428
La	0.927941	0.924245	0.927732	71.72904	44.34606	72.82573
ElNe	0.927941	0.924244	0.927732	71.72819	44.34567	72.825
XGBo	0.927941	0.924245	0.927732	71.7288	44.34558	72.82477
LiGBM	0.999994	0.999451	0.999506	0.40228	4.838782	5.891523
RaFo	0.999899	0.999715	0.9997	3.760146	4.514523	5.63265

**Fig. 1:**
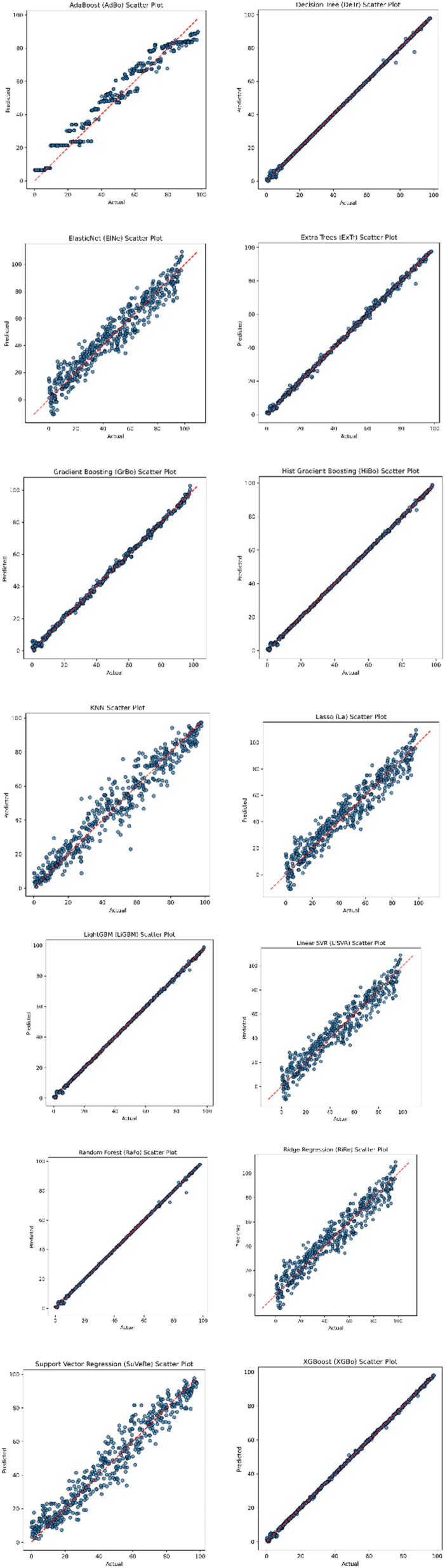
Scatter plot of all the applied ML algorithms.

It identified five top-performing algorithms on the basis of their comparative performance: RaFo, ExTr, GrBo, HiBo, and XGBo. These models reached the best *R*^2^ values, the lowest prediction errors, and smooth residual distributions across validation folds. Their dominance confirmed that approaches of ensemble learning are effectively applicable for high-dimensional environmental reaction datasets. Next, selected top models were used for hybrid model development with the aim to enhance the accuracy and stability of predictions. Fig. 1 shows the scatter plot of all the applied ML algorithms generated from google colab (one algorithm did not respond which has been excluded).

### Hybrid model development using top 5 algorithms

**Fig. 2:**
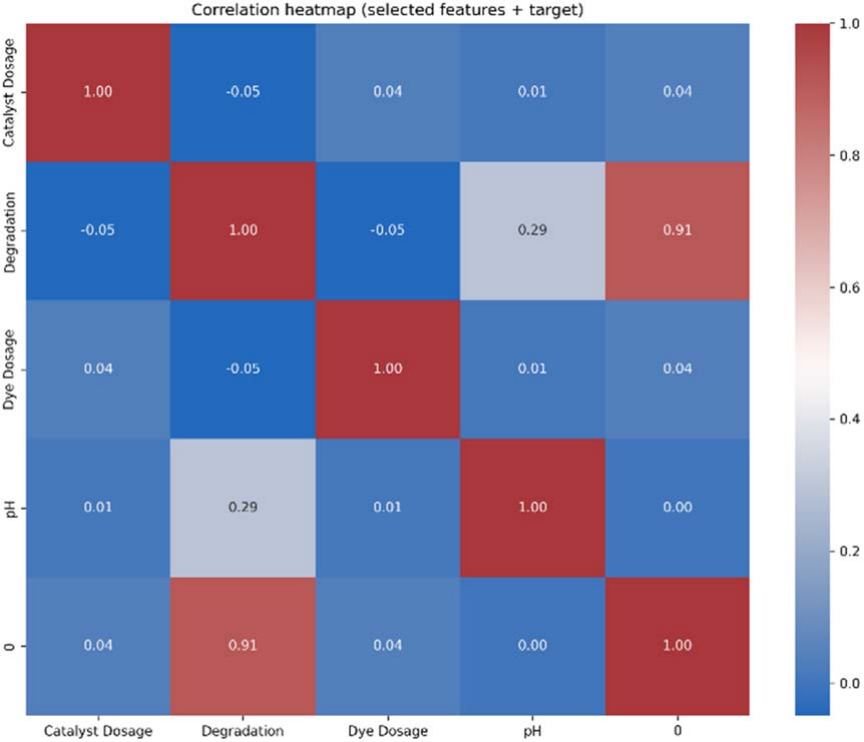
Correlation heatmap.

Hybrid models were generated based on pairwise fusion across the top-five highest-performance algorithms. Each of the possible pairs among algorithms like RaFo, ExTr, GrBo, HiBo, and XGBo have been hybridized using weighted averaging and stacked ensemble mechanisms. In this way, ten hybrid combinations in total were created and tested. The rationale behind doing so is to combine the strengths of the individual models and reduce prediction variance due to the limitations that come with using isolated algorithms. Weighted averaging assigns proportional dominance to the models based on their comparative accuracies, while stacking algorithms depend on a secondary meta-learner for refinement of the final predictions. Fig. 2 shows the correlation heatmap and the detailed explanation of correlation heatmap as well as all the results parameters and the experimental parameters have been included in SI document.

**Fig. 3:**
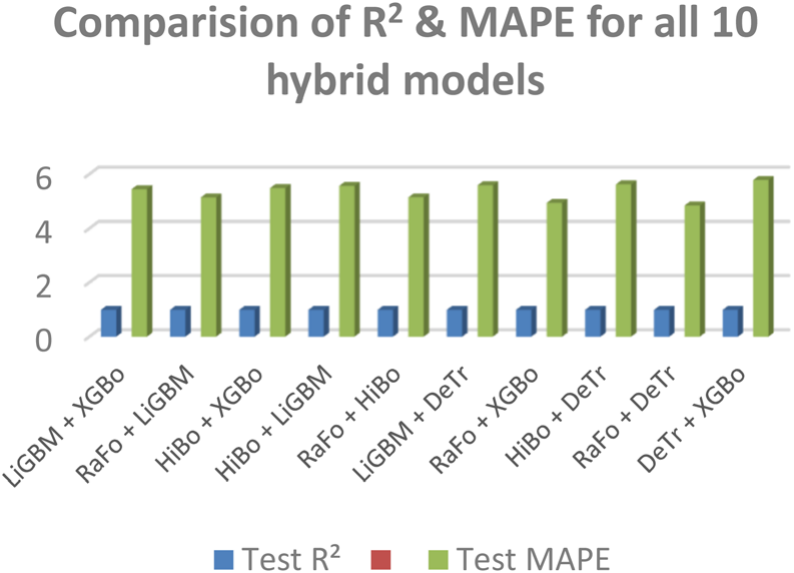
Comparison of 10 hybrid models.

Among all tested hybrids, RaFo + HiBo (RFHGB) performed the best, providing the best accuracy in degradation predictions along with minimum MAE (Fig. 3). RFHGB outperformed all other combinations due to complementary learning behavior, where RaFo handled high-dimensional feature variation effectively, and the improvement pattern based on gradients was effectively captured by HiBo. In support, the parity plots and residual distribution curves showed that actual and predicted values are in close agreement with each other, which reflects great fitting capability. Thus, RFHGB was found to be the best hybrid model for catalyst performance prediction and operational optimization.

### Catalyst ranking and prediction of optimal conditions

Catalytic performance ranking was, therefore, generated for the most effective degradation conditions across SrO, and synthetic compositions of ZnO–CuO using the best performing RFHGB hybrid model. The ZnO–CuO catalyst ranked first in predicted degradation efficiency, confirming its experimentally observed superior performance. Machine learning predictions of optimal settings of parameters yielded that a moderate catalyst dosage, slightly acidic to neutral pH, higher light intensity, and extended irradiation time gave the maximum degradation performance. Similarly, the model predicts some hypothetical catalyst formulations within the domain of the synthetic dataset, allowing one to glean some idea about potential improvements by further doping or compositional modification strategies.

These predictions pinpoint machine learning as a valuable decision-making tool to guide catalyst design, reduce experimental costs, and accelerate material discovery. The capability for reliable predictions of degradation behavior allows the researcher to explore greater experimental possibilities without requiring extensive effort in the laboratory. Model interpretability studies using SHAP analysis further revealed that pH, irradiation time, catalyst dosage, and band-gap energy were the most influential parameters controlling degradation efficiency.

#### Data distribution & feature analysis figures

##### Distribution comparison: histograms, KDEs, boxplots, violin plots

**Fig. 4:**
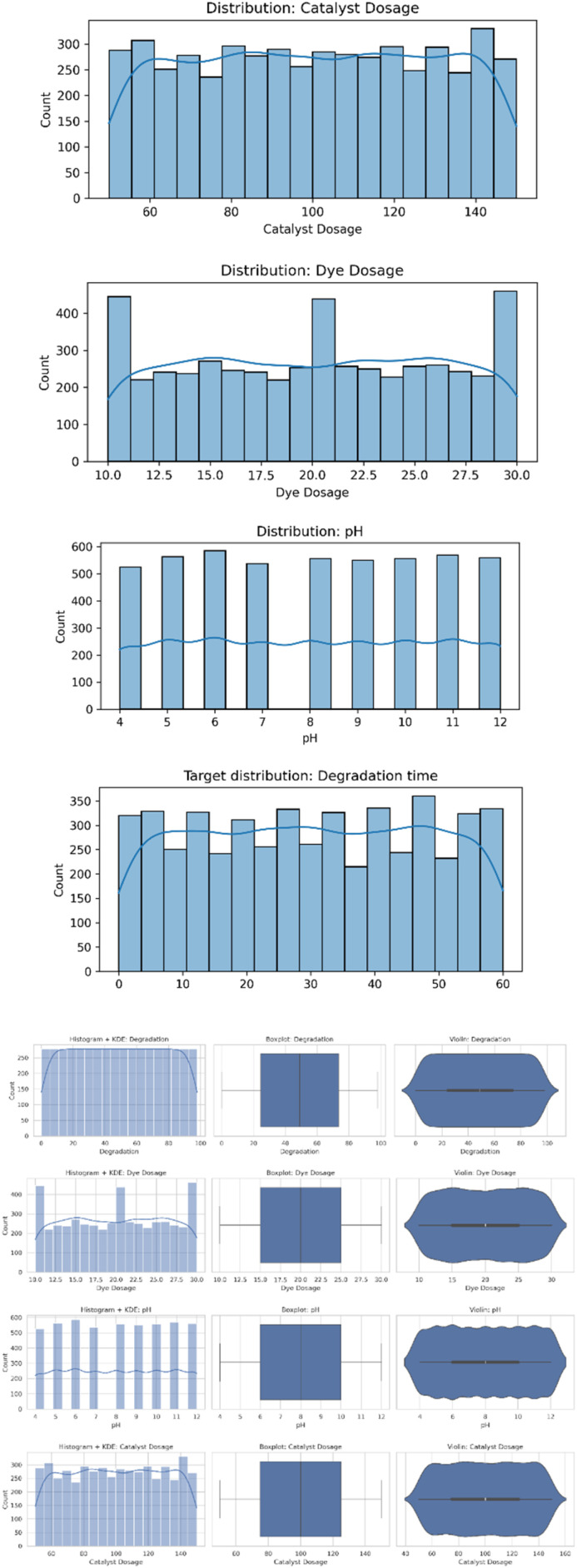
Distribution comparison: histograms, KDEs, boxplots, violin plots for all the taken parameters such as dye dosage, catalyst dosage, pH, degradation time.

The distribution plots offer a deep view of each feature's statistical behavior within the dataset (Fig. 4). Histograms and KDE curves give insight into the shape, skewness, and spread of the features' distributions, possibly highlighting outliers, non-linearity, and multimodal behavior. Complementary boxplots and violin plots help to emphasize central tendencies, dispersions, and extreme values. This will make sure that the dataset is well-characterized in advance of model training and will help guide the selection of normalization or transformation strategies.

##### Pair-plot

**Fig. 5:**
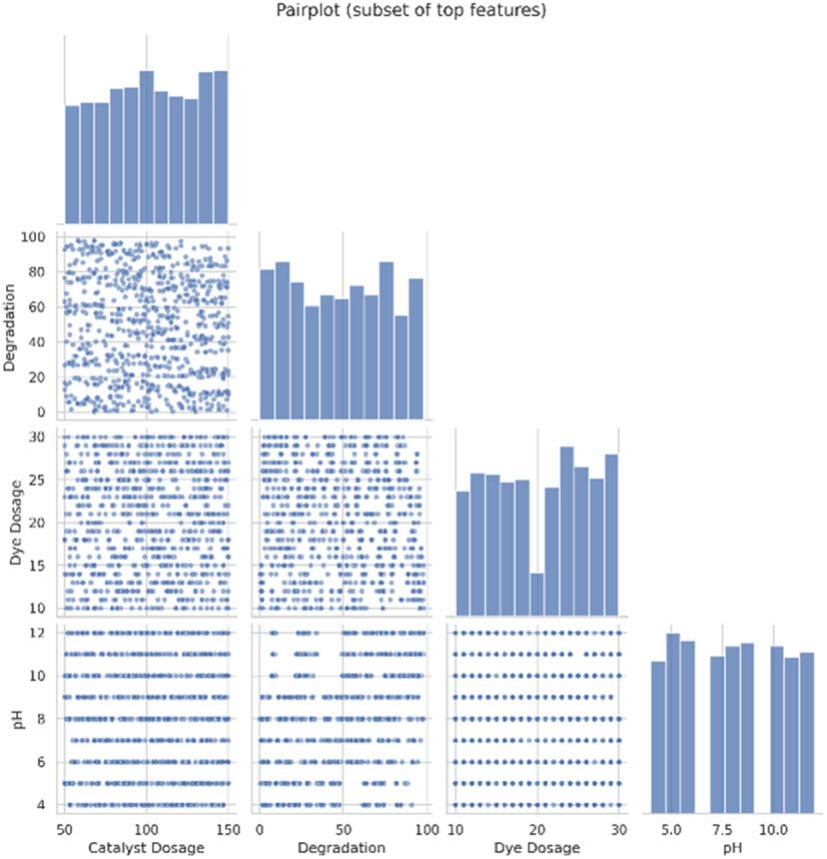
Pair plot for the synthetic dataset of 5000 datas.

The pair plot (Fig. 5) visualizes pairwise relationships among the most informative features in the dataset. It shows possible correlations, nonlinear interactions, and clustering behavior that may affect model performance. The diagonal density plots display the individual feature distributions, while the scatterplots show how features vary jointly. This multi-feature visualization helps to assess whether the dataset contains redundant variables or complex interactions that require nonlinear approaches to modelling.

##### PCA/UMAP scatter plots (overlap & data structure)

**Fig. 6:**
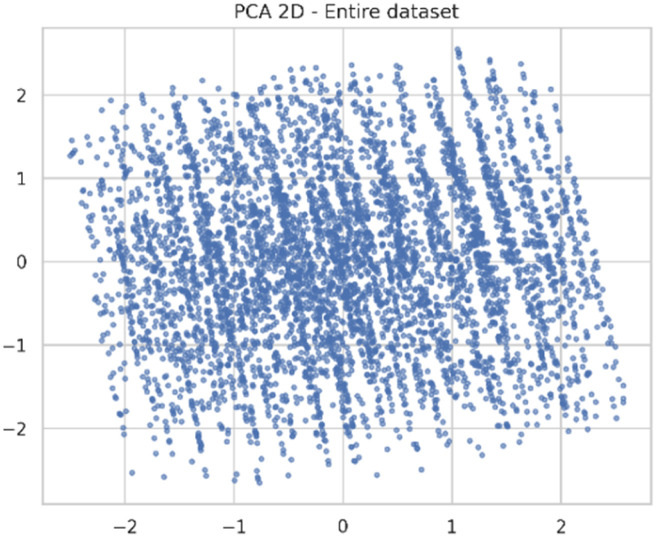
PCA scatter plot for dimensionality reduction analysis.

Dimensionality reduction plots including PCA and UMAP allow for the inspection of global dataset structure by mapping high-dimensional features onto a low-dimensional manifold. While PCA captures the most variance in linear components, UMAP models non-linear relationships between them (Fig. 6). The resulting 2D embeddings present insights into sample scattering, highlight hidden clusters, and allow the checking of whether the dataset is variable enough to allow training robust models. These plots confirm the diversity of the representations within the synthetic dataset. The modeling task was identified as the prediction of the efficiency of the degradation of PRS as a function of various operational parameters such as the amount of catalyst used, the pH of the medium, time of irradiation, and concentration of the dye used. The pH of the medium was treated as a continuous variable within the modeling framework. Although a specific set of experiments was conducted at pH = 7 for relevance to the environment, the prediction task was not limited to that specific case.

#### Synthetic data validation figures

**Fig. 7:**
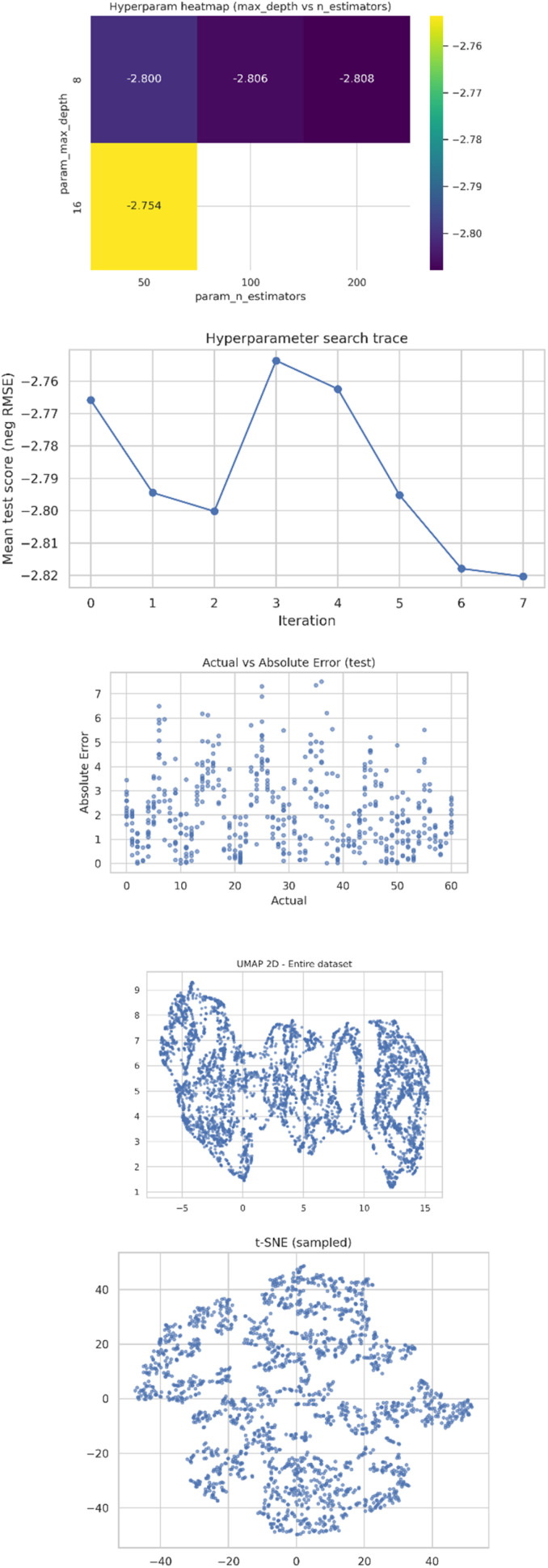
Analysis of t-SNE, UMAP and error analysis.

The various synthetic data validation plots assess the fidelity and quality of generated data. Variations in PCA and t-SNE visualizations establish if the samples fall within a realistic and coherent region in feature space, which further supports that the synthetic points maintain the geometrical structure naturally. For a generative model like GAN or VAE, the loss-curve plot monitors the reconstruction and divergence behavior over epochs to ensure stable model convergence. Divergence metrics and distance-based evaluations, upon visualization, further ensure that the synthetic data falls statistically in line with expected distributions and introduce no bias. Fig. 7 shows the plots generated from the analysis of t-SNE, UMAP and error analysis. The plots of hyperparameter optimization show the behavior and convergence of the model for RandomizedSearch and GridSearch as shown below (google colab output).
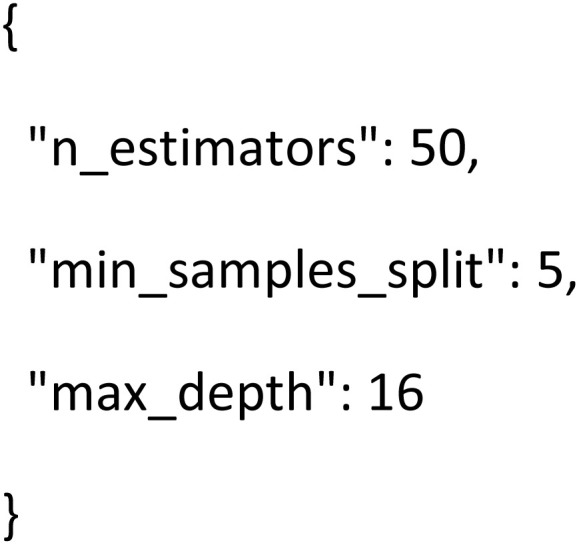


An optimization trace plot provides information about improved model performance through successive iterations, indicating the efficacy of the search algorithm in traversing the space of parameters. Heatmaps of hyperparameter combinations detail performance gradients with respect to depth *versus* number of estimators, learning rate, or regularization parameters. Such plots allow for the identification of optimal configurations, hence supporting a rationalized choice of the best-performing model.

#### SHAP summary and SHAP dependence plots

**Fig. 8:**
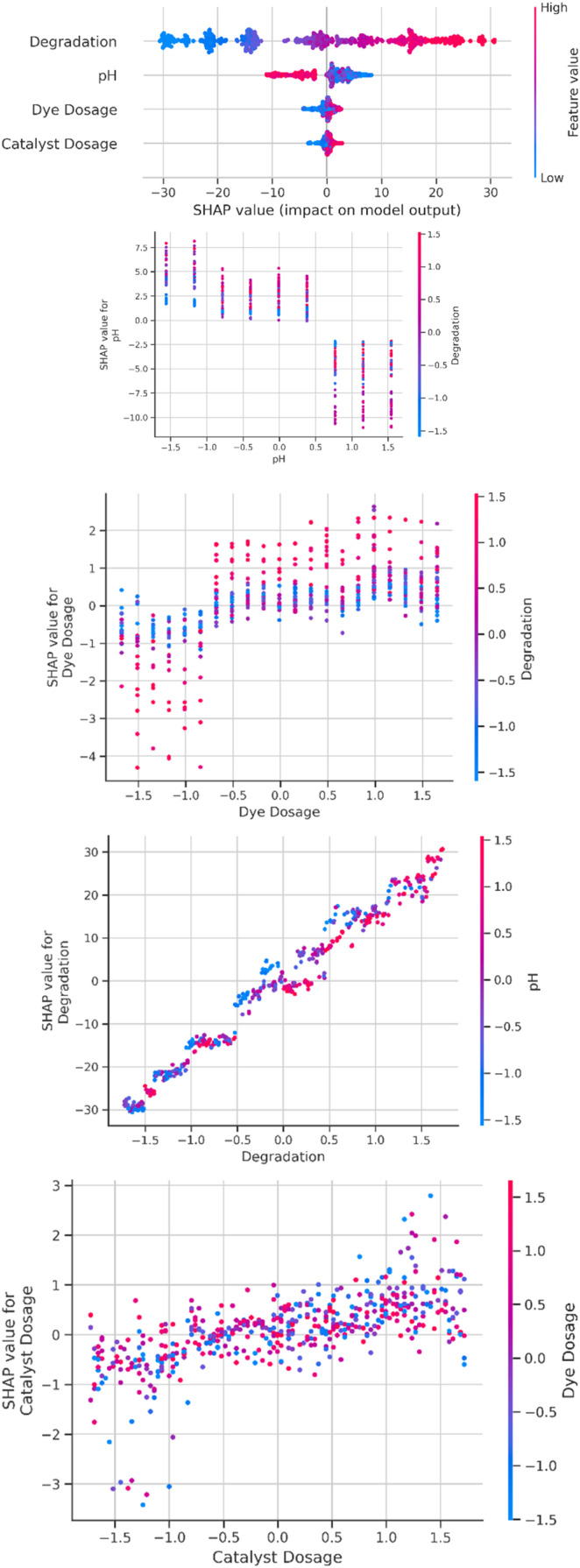
SHAP plots (overall and separately for all the parameters).

SHAP explainability plots quantify and visualize the feature contributions to the model's predictions. The SHAP summary plot (Fig. 8) ranks features in terms of their overall impact and illustrates the direction of their influence relative to the target variable. Dependence plots afford deeper insights by showing how changes in a particular feature will affect the prediction, while highlighting interactions with other variables. These visualizations, put together, provide a transparent interpretation of the model decision-making process that pinpoints the most important physicochemical variables governing degradation efficiency.

#### Error analysis figures

**Fig. 9:**
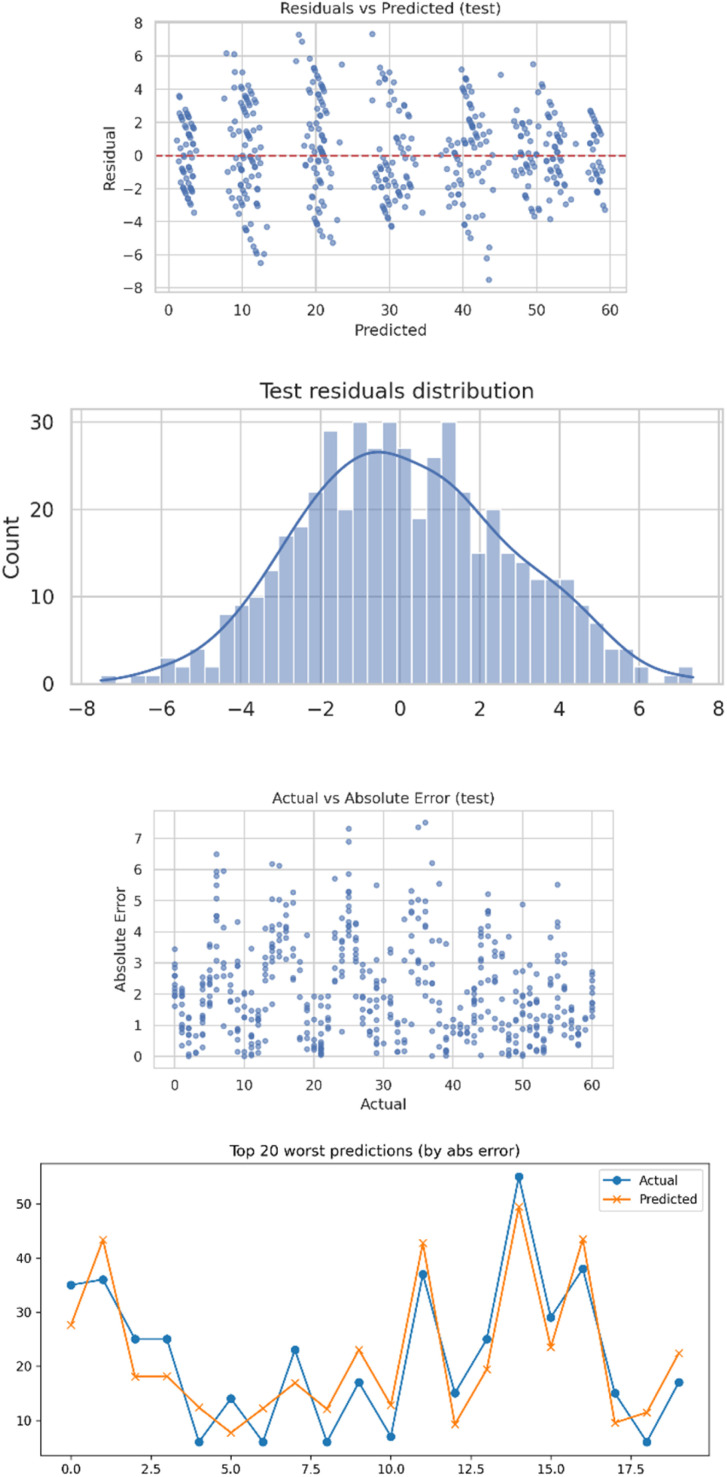
Error analysis for 5000 datasets.

Error analysis visualizations help diagnose model reliability and find failure patterns (Fig. 9). The residual distribution histogram presents deviation from the ideal zero-error behavior, and it could highlight skewness or heavy tails (Table 2). Scatter plots of residuals *versus* predictions detect heteroscedasticity or systematic bias, while actual *versus* error plots quantify the magnitude of prediction errors across different target levels. Collectively, these figures ensure model consistency in behavior for the full range of its predictions and provide evidence of its robustness.

**Table 2:** Worst 20 errors from absolute error analysis

Rank	Actual	Predicted	Abs error
1	36	43.51071	7.510706
2	35	27.64886	7.351143
3	25	17.68935	7.310651
4	25	18.11125	6.888748
5	6	12.49245	6.492449
6	37	43.20271	6.202714
7	14	7.817222	6.182778
8	15	8.876175	6.123825
9	7	12.94772	5.947722
10	6	11.92815	5.928151
11	25	19.14865	5.851349
12	6	11.78576	5.785761
13	23	17.29925	5.700746
14	38	43.54523	5.54523
15	55	49.48543	5.514571
16	6	11.49882	5.498817
17	29	23.5012	5.498802
18	34	28.6883	5.311697
19	25	19.70335	5.296649
20	17	22.27181	5.27181

#### Limitations and prospective

• Dependence on the studied catalysts (ZnO–CuO and SrO).

• Generalizability of the trained model to new catalysts.

• Need for future integration of catalyst physicochemical descriptors.

• Expansion toward larger, multi-source experimental datasets.

This addition provides a balanced outlook without altering the core conclusions of the study.

## Conclusion

This study demonstrates that integrating machine learning with experimental photocatalysis provides an effective pathway for predicting and optimizing catalyst performance for PRS pollutant degradation. In the present work, two photocatalysts, ZnO–CuO (synthesized) and SrO (literature), were considered and their photocatalytic efficiencies were evaluated experimentally, thereby developing an initial dataset of 81 observations. By applying computational synthetic data augmentation, the dataset was increased to 5000 points, which allowed for deeper learning and generalized behavior of the model. To establish a baseline in performance, a total of 15 machine learning algorithms were trained, out of which the top five models were selected for hybridization based on the evaluation metrics of *R*^2^, RMSE, and MAE. The best hybrid model was identified as the RaFo + HiBo combination, which showed exceptional predictive accuracy and reliable correlation between actual and predicted values.

The hybrid approach presented here, in fact, successfully ranked the catalysts and identified ZnO–CuO as the most efficient material under optimized operating conditions. This demonstrates that the capability of the machine learning framework in capturing nonlinear relationships between operational variables and degradation outcomes could play a decisive role in environmental process optimization using computational tools. While the methodology presented herein reduces experimental workloads and associated costs, it accelerates the discovery of advanced materials for emerging pollutant treatment. In summary, this work illustrates the potential of using data-driven models as a guide for designing catalysts with the objective of developing sustainable solutions for wastewater treatment.

PRS represents only one member contamination family, testing the model against structurally different pollutants such as methylene blue, malachite green, and crystal violet can strengthen the universality of the predictive platform. Future work can be directed toward multi-metal oxide catalysts, doped or co-doped hybrid nanostructures that adopt both plasmonic and semiconductor properties to realize more efficient visible-light-driven photocatalysis. Real-scale reactor modelling and associated pilot-plant experiments would also provide further verifications of feasibility beyond laboratory-scale batch systems.

Moreover, with the integration of real-time reactor monitoring and closed-loop feedback control using IoT sensors and machine learning algorithms, an intelligent wastewater treatment system may achieve continuous optimization. The data can be enriched by high-throughput experimentation and the coupling of machine learning to molecular simulation tools such as DFT or MD for atomic-level insight into charge behavior and surface-active site interactions. Several techniques that might be used to improve trust in AI-supported decisions include explainable AI approaches such as SHAP interpretation and LIME visualization. Ultimately, it will be through theory, experiment, and computation that commercially scalable energy-efficient photocatalytic treatment technologies will be developed.

In comparing the performance of all models, model comparisons were conducted under a unified framework. Although several models were found to have comparable levels of predictive accuracy, relatively improved performance was noted for the Random Forest–Histogram Gradient Boosting hybrid.

## Author contributions

Anandhi: conceptualization, data curation, investigation, software, formal analysis, writing – original draft. Iyapparaja: conceptualization, writing – review and editing, validation, supervision.

## Conflicts of interest

The authors declare no relationships or financial interests that could have influenced the study.

## Supplementary Material

RA-016-D5RA09598K-s001

## Data Availability

All data supporting the findings of this research study are available within the article and its supplementary information (SI). Additional datasets (synthetic data) are available from the corresponding author on reasonable request. Supplementary information: all the mathematical expressions, google colab link, as well as experimental section performed by the authors. See DOI: https://doi.org/10.1039/d5ra09598k.
