# Supplementary material for: Machine learning driven identification of optimal nanomaterials for efficient pararosaniline dye removal from water using a RFHGB hybrid model
Source: RSC Adv. 2026 Mar 4;16(14):12315–25. doi: 10.1039/d5ra09598k (PMC12959327; doi:10.1039/d5ra09598k)
Supplement: RA-016-D5RA09598K-s001 [file RA-016-D5RA09598K-s001.pdf]

Supplementary Information (SI) for RSC Advances.
This journal is © The Royal Society of Chemistry 2026

Machine Learning Driven Identification of Optimal Nanomaterials for Efficient Pararosaniline Dye Removal from Water Using a RFHGB Hybrid Model

Ganesan Anandhi, M. Iyapparaja*

Department of Smart Computing, School of Computer Science Engineering and Information Systems, Vellore Institute of Technology, Vellore 632014, Tamil Nadu, India.

*Corresponding author: E-mail: iyapparaja.m@vit.ac.in, Ph.: +919942532800

Supplementary Document

1. The google colab link has been share below for the additional reference purpose:

https://colab.research.google.com/drive/1LJvBw8chve6MfQj3etiCZhlHPbtQY_EN

2. Snippet code

```
# -------------------------- IMPORTS --------------------------
import pandas as pd
import numpy as np
import warnings
from itertools import combinations
from sklearn.model_selection import train_test_split
from sklearn.metrics import (
    mean_absolute_error, mean_squared_error, r2_score,
    explained_variance_score, mean_absolute_percentage_error
)
from sklearn.ensemble import RandomForestRegressor, ExtraTreesRegressor,
StackingRegressor
from sklearn.tree import DecisionTreeRegressor
from sklearn.linear import BayesianRidge
from sklearn.base import clone
import matplotlib.pyplot as plt

warnings.filterwarnings("ignore")

# Optional models
try:
    from xgboost import XGBRegressor; HAS_XGB = True
except: HAS_XGB = False
```

```
try:
    from lightgbm import LGBMRegressor; HAS_LGBM = True
except: HAS_LGBM = False


# --------------------------- LOAD DATA ---------------------------
df = pd.read_excel("data5000.xlsx")
target = df.columns[-1]

# Filter pH = 7
df = df[df["pH"] == 7].copy()


# --------------------------- FEATURES / TARGET ---------------------------
X = pd.get_dummies(df.drop(columns=[target]), drop_first=True)
y = df[target]

# Train/Val/Test split
X_train_val, X_test, y_train_val, y_test = train_test_split(X, y, test_size=0.10, random_state=42)
X_train, X_val, y_train, y_val = train_test_split(X_train_val, y_train_val, test_size=0.20, random_state=42)


# --------------------------- METRICS FUNCTION ---------------------------
def get_metrics(y_true, y_pred, Xd):
    mae  = mean_absolute_error(y_true, y_pred)
    mse  = mean_squared_error(y_true, y_pred)
    rmse = np.sqrt(mse)
    r2   = r2_score(y_true, y_pred)
    n, p = len(y_true), Xd.shape[1]
    adjr2 = 1 - (1 - r2) * (n - 1) / max(n - p - 1, 1)
    mape = mean_absolute_percentage_error(y_true, y_pred) * 100
    evs  = explained_variance_score(y_true, y_pred)
    return mae, mse, rmse, r2, adjr2, mape, evs


# --------------------------- BASE MODELS ---------------------------
base_models = {
    "Random Forest": RandomForestRegressor(n_estimators=200, random_state=42),
    "Extra Trees": ExtraTreesRegressor(n_estimators=200, random_state=42),
    "Decision Tree": DecisionTreeRegressor(random_state=42)
}
```

```
if HAS_LGBM: base_models["LightGBM"] = LGBMRegressor(n_estimators=250,
random_state=42)
if HAS_XGB: base_models["XGBoost"] = XGBRegressor(n_estimators=250,
random_state=42, verbosity=0)

models = {f"Base - {k}": v for k, v in base_models.items()}

# Hybrid pairs
for (n1, m1), (n2, m2) in combinations(base_models.items(), 2):
   models[f"Hybrid - {n1} + {n2}"] = StackingRegressor(
      estimators=[(n1, clone(m1)), (n2, clone(m2))],
      final_estimator=BayesianRidge(), n_jobs=-1
   )


# --------------------------- TRAIN + COLLECT METRICS ---------------------------
results = []
for name, model in models.items():
   model.fit(X_train, y_train)

   y_tr = model.predict(X_train)
   y_v  = model.predict(X_val)
   y_te = model.predict(X_test)

   tr = get_metrics(y_train, y_tr, X_train)
   v  = get_metrics(y_val, y_v, X_val)
   te = get_metrics(y_test, y_te, X_test)

   results.append([name, tr[3], v[3], te[3], tr[5], v[5], te[5], tr[0], v[0], te[0]])

cols = ["Model","Train R²","Val R²","Test R²","Train MAPE","Val MAPE","Test
MAPE","Train MAE","Val MAE","Test MAE"]
df_results = pd.DataFrame(results, columns=cols)


# --------------------------- SELECT BEST MODELS ---------------------------
base_df = df_results[df_results.Model.str.contains("Base")]
hybrid_df = df_results[df_results.Model.str.contains("Hybrid")]

best_base = base_df.sort_values(["Test R²","Val MAPE"],
ascending=[False,True]).iloc[0]
```

```
best_hybrid = hybrid_df.sort_values(["Test R²","Val MAPE"], ascending=[False,True]).iloc[0]

best_hybrid_model = models[best_hybrid["Model"]]


# --------------------------- APPLY BEST HYBRID ON ALL pH=7 ---------------------------
y_pred_all = best_hybrid_model.predict(X)
df_all = df.copy()
df_all["Actual"] = y
df_all["Pred"] = y_pred_all
df_all["APE %"] = np.abs((df_all["Actual"] - df_all["Pred"]) / df_all["Actual"]) * 100

df_all.to_excel("best_conditions_pH7.xlsx", index=False)


# --------------------------- PLOT: ACTUAL vs PREDICTED ---------------------------
plt.figure(figsize=(6,6))
plt.scatter(df_all["Actual"], df_all["Pred"], s=180, alpha=0.55, edgecolor="black")
mn, mx = min(df_all["Actual"].min(), df_all["Pred"].min()), max(df_all["Actual"].max(), df_all["Pred"].max())
plt.plot([mn, mx], [mn, mx], "--", linewidth=0.8, color="black")

plt.xlabel("Actual (pH = 7)")
plt.ylabel("Predicted")
plt.title(f"Actual vs Predicted — Best Hybrid Model\n{best_hybrid['Model']}")
plt.tight_layout()
plt.savefig("Actual_vs_Predicted_pH7.png", dpi=300)
plt.show()
```

3. Machine Learning formulas

Random Forest: Random Forest is an ensemble learning method that builds multiple decision trees on random subsets of data and features. The final prediction is the average of all tree outputs (for regression).

Prediction $\hat{y}$ is given by: $\hat{y} = \frac{1}{T}\sum_{t=1}^{T} f_t(x)$

Where $T = number\ of\ trees,$ and $f_t(x) = prediction\ of\ t$

Example: If three trees predict: 80, 90, 85 then Prediction = (80 + 90 + 85) / 3 = **85**.

Extra Trees: Extra Trees is similar to Random Forest but uses fully random splits for feature thresholds, increasing diversity.

$$\hat{y} = \frac{1}{T}\sum_{t=1}^{T} f_t(x)$$

Decision Tree: A Decision Tree splits the dataset into regions and assigns each region the average of the target values inside it.

If a point x belongs to a region R_j:

$$\hat{y} = \frac{1}{|R_j|}\sum_{x_i \in R_j} y_i$$

AdaBoost: AdaBoost builds weak learners sequentially, giving more weight to samples with high error.

$$\hat{y} = \sum_{t=1}^{T} \alpha_t f_t(x)$$

Where, α_t is the weight of week learner

Example: If $f_1(x)=50$, $f_2(x)=60$ with weights $\alpha1=0.4$, $\alpha2=0.6$

Prediction = 0.4(50) + 0.6(60) = **56**.

Gradient Boosting: Gradient Boosting trains weak learners sequentially, each minimizing the previous model's residual (error).

$$F_m(x) = F_{m-1}(x) + \gamma_m h_m(x)$$

Where $h_m(x)$ is a weak learner on residuals.

Example: Initial predication=50, Residual = true(70) - predicted(50)=20, If weak learner corrects 70% → update = 50 + 0.7×20 = 64.

Histogram Gradient Boosting: A faster version of Gradient Boosting that bins continuous features into histograms before building trees.

$$F_m(x) = F_{m-1}(x) + \gamma_m h_m(x)$$

Example: Bins: 10–20, 20–30, 30–40
Sample falls in 20–30 bin with residual +15
Update = previous 55 + 0.6×15 = **64**.

K-Nearest Neighbours (KNN): KNN predicts the value of a point by averaging its k nearest neighbours.

$$\hat{y} = \frac{1}{K}\sum_{i=1}^{k} y_i$$

***Example*:** Neighbours: 70, 75, 80 | Prediction = (70+75+80)/3 = **75**.

Support Vector Regression (SVR – RBF kernel, scaled): SVR finds a function that fits within a margin ε while minimizing model complexity.

Prediction:

$$\hat{y} = \sum_{i=1}^{n} (\alpha_i - \alpha_i)K(x_i, x) + b$$

RBF Kernel:

$$K(x_i, x) = exp(-\gamma\|x_i - x\|^2)$$

Example: If α difference=2, kernel=0.5, b=10 →
Prediction = 2×0.5 + 10 = **11**.

Linear SVR (scaled): A linear version of SVR using linear kernel.

$$\hat{y} = w^T x + b$$

Example: If w = [2,1], x = [3,5], b = 4 →
Prediction = 2×3 + 1×5 + 4 = **15**.

Kernel Ridge: Combines Ridge Regression with kernel trick for non-linear fitting.

$$\hat{y} = K(K + \lambda I)^{-1} y$$

Example:
Kernel matrix K = [[1,0.5],[0.5,1]]
λ = 1
Compute prediction for y=[10,20] → approx. **14.3**

Linear Regression: Fits a straight line that minimises squared errors.

$$\hat{y} = w^T x + b$$

Example:
If w=[3], x=4, b=2 →
Prediction = 3×4 + 2 = **14**.

Bayesian Ridge: Probabilistic version of Ridge Regression estimating distribution over coefficients.

Posterior mean of weights: $w = (X^T X + \lambda I)^{-1} X^T y$

Example: Same matrix as Ridge → estimated w reduces slightly for regularisation.

Ridge Regression: Linear regression with L2 penalty to control overfitting.

$$w = (X^T X + \lambda I)^{-1} X^T y$$

Example:
If λ = 1 reduces coefficient from 4.0 → **3.7**.

Lasso Regression: Linear regression with L1 penalty causing feature selection (coefficients shrink to zero).

$$\min_w (\|y - Xw\|_2^2 + \lambda_1 \|w\|_1)$$

Example: Coefficient reduces from 4.0 → becomes **0** if penalty is high.

ElasticNet Regression: Combines Lasso (L1) and Ridge (L2) penalties.

$$\min_w (\|y - Xw\|_2^2 + \lambda_1 \|w\|_1 + \lambda_1 \|x\|_2^2)$$

Example: Coefficient 4.0 becomes **1.8** (part shrinkage, not zero).

4. Literature Review data (IEEE data port hyperlink)
https://ieee-dataport.org/documents/photocatalytic-degradation-pararosaniline-nano-sro-experimental-data

5. Experimental Procedure and Results

Materials and Method

Materials

Copper(II) chloride dihydrate ($CuCl_2 \cdot 2H_2O$, ≥99% purity), zinc nitrate hexahydrate ($Zn(NO_3)_2 \cdot 6H_2O$, ≥99% purity), and sodium hydroxide (NaOH, pellets, ≥98% purity) were procured from Sigma-Aldrich and used without further purification. PRS dye (analytical grade) was purchased from SRL Chemicals (India), Ethanol and Distilled Water (DIW). All chemicals were of analytical grade unless otherwise specified.

Synthesis of CuO and ZnO

The CuO and ZnO was prepared by using simple hydrothermal reaction. Firstly, 10 mm of $CuCl_2 \cdot 2H_2O$ was added to 50 ml of DIW and Kept for stirring. Secondly, add 2 M NaOH solution drop by drop until the solution reach pH 12 and keep stirring for 1 h at 60 ^oC. After that take solution for centrifuge with DIW and Ethanol for 30 mins to remove impurities and

keep drying for 24 h for 70 °C at Hot air oven. Then keep the product in muffle furnace at 400 °C for 3 h. Receptivity, instead of $CuCl_2·2H_2O$ replace with $Zn(NO_3)_2·6H_2O$ to from a ZnO.

Synthesis of ZnO-CuO nanocomposite

The ZnO-CuO was prepared by using ultrasonication method. The equal weight of CuO and ZnO was added to the 50 ml of Ethanol:DIW (1:1) and keep for probe sonication for 40% amplitude, pulsed 5s ON/5s OFF, total ON time 30 min. After that take solution for centrifuge with DIW and Ethanol for 30 mins to remove impurities and keep drying for 24 h for 70 °C at Hot air oven. Then keep the product in muffle furnace at 400 °C for 3 h and collect the final ZnO-CuO nanocomposite.

Photocatalytic degradation experiment of PRS dye

The photocatalytic activity of the synthesized ZnO-CuO was evaluated by measuring the degradation of PRS dye under visible light irradiation. In each experiment, 50 mL of a 13, 10, 14 ppm aqueous dye was combined with 65, 56, 73 mg respectively of ZnO-CuO into a reactor. To reach adsorption-desorption equilibrium, the suspension was magnetically stirred for 30 min without light before illumination. After that, the reactor was exposed to visible light (240 W Tungsten lamp), and aliquots were taken for analysis every ten minutes. Spectrophotometric measurements of the residual levels of PRS were made at 540 nm.

Results and Discussion

XRD

The XRD were utilized for investigate the crystalline structure, with the configurations of ZnO-CuO heterojunction presented in Fig.1S. The diffraction peaks the CuO phase was identified from the $2\theta = 32.5^{\circ}$, 35.5°, 38.7°, 48.7°, 53.3° and 61.5° is corresponds to the planes respectively (110), (–111), (111), (–202), (020), and (–113) well matched with JCPDS No. 45-0937. In addition, ZnO were clearly observed at $2\theta = 31.7^{\circ}$, 34.4°, 36.2°, 47.5°, 56.6° and 62.8° is corresponding the plane respectively (100), (002), (101), (102), (110), and (103) are matched with JCPDS No. 36-1451. The presence of both sets of peaks confirms the successful formation of a ZnO-CuO heterojunction. The slight broadening of the diffraction peaks indicates the nanoscale nature of the particles, while the coexistence of CuO and ZnO peaks without any impurity phases demonstrates good crystallinity and proper incorporation of ZnO onto the CuO surface. These features are favorable for enhanced photocatalytic performance due to improved charge separation at the CuO–ZnO interface.

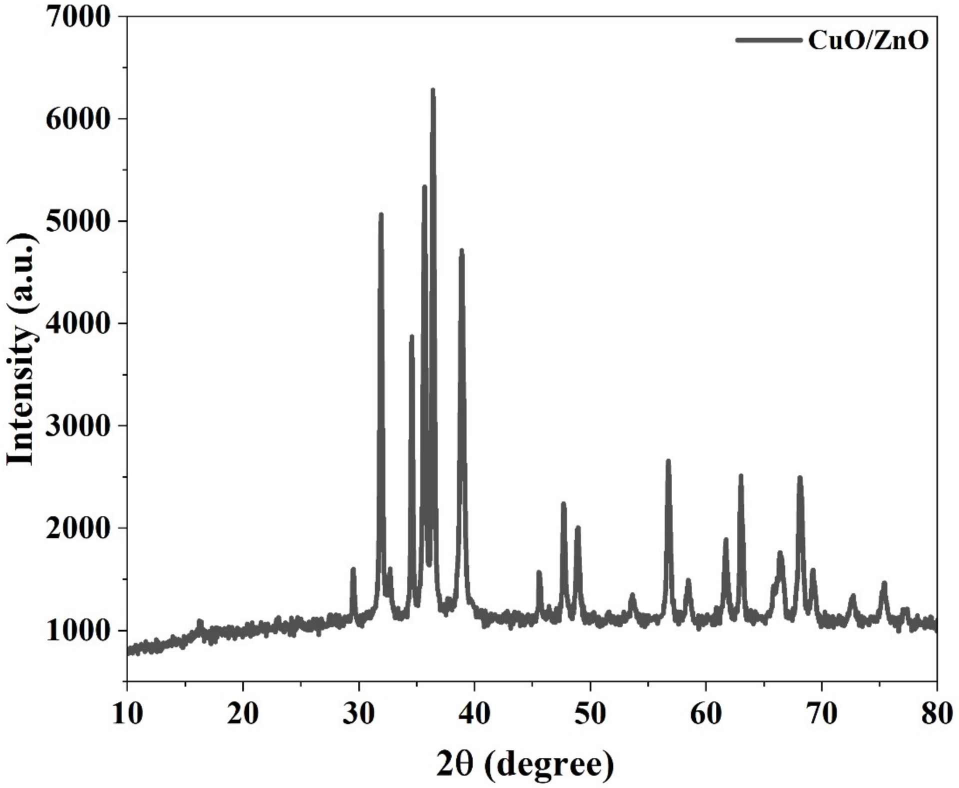

Fig.1S XRD pattern of ZnO-CuO

UV–vis DRS spectroscopy

UV–Vis DRS was used to analyze the ZnO-CuO nanocomposite light absorption characteristics (Fig. 2(a)S). The composite exhibits a broad absorption profile covering the entire 200–800 nm range, demonstrating its capability to both UV and visible light. The strong absorption edge observed in the UV region (250–380 nm) is attributed to intrinsic band-to-band transitions in ZnO, which possesses a wide band gap (~. In contrast, the extended absorption tail in the visible region originates from CuO, a narrow-band-gap p-type semiconductor that absorbs strongly in the 450–800 nm region. The merged absorption features confirm the successful coupling of CuO with ZnO and indicate enhanced optical activity due to interfacial interaction between the two oxide phases. The corresponding Tauc plot (Fig. 2(b)S) was used to estimate the effective band gap, which was found to be 2.80 eV for the composite. This value lies between those of pure ZnO and CuO, indicating band-gap modulation caused by charge transfer and electronic coupling at the ZnO-CuO interface. The slight narrowing of the band gap suggests improved visible-light utilization and easier electron excitation.

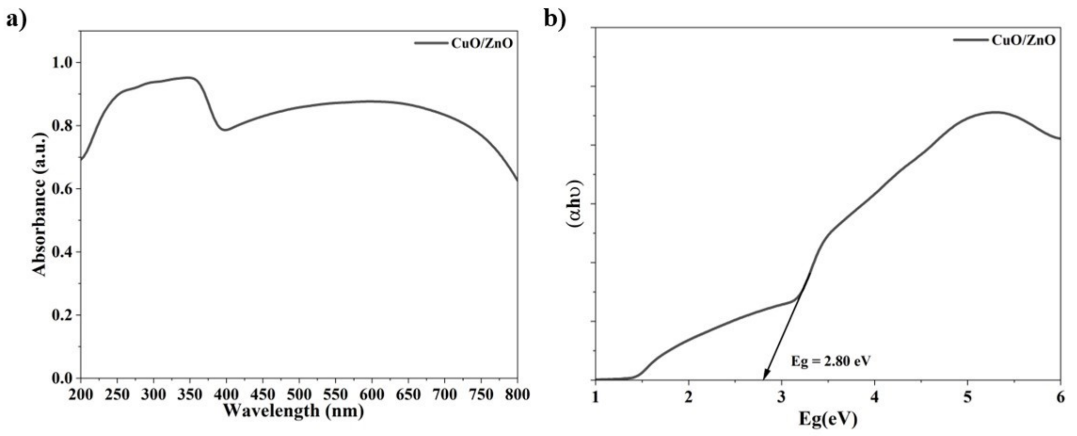

Fig. 2(a)S UV–vis DRS spectra 2(b)S Band gap of ZnO-CuO nanocomposite

Morphological aspect

The surface morphology of the samples was analyzed using SEM, as shown in Fig. 3S(a-b). The pristine CuO displays agglomerated, porous clusters resembling flower-like nanostructures, which is typical of solution-processed CuO. These highly rough and irregular aggregates offer a large surface area and adsorption sites. Upon coupling with ZnO, a distinct change in morphology is observed. The ZnO-CuO nanocomposite appears denser and more compact, with finer nanoparticles uniformly distributed over the CuO surface. The presence of ZnO particles decorating the CuO matrix confirms the successful formation of a heterojunction structure. This modified morphology enhances interfacial contact between the two semiconductors, which is crucial for facilitating charge separation and reducing electron-hole recombination.

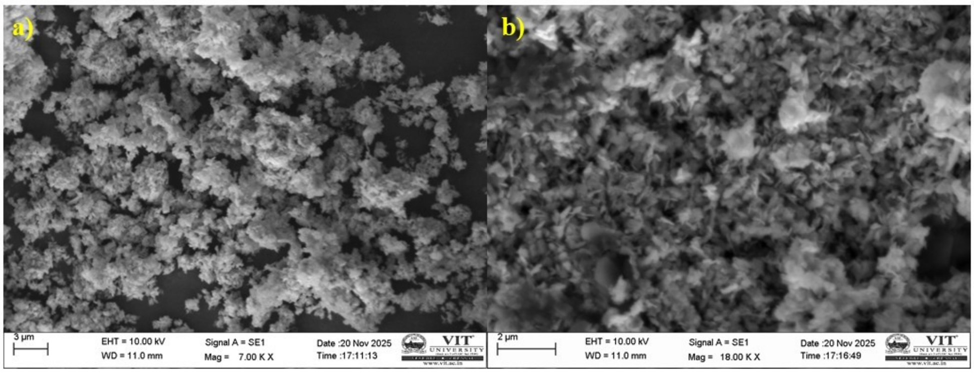

Fig. 3S SEM image of ZnO-CuO nanocomposite

The EDAX and mapping of ZnO-CuO nanocomposite was shown in Fig. 4S. The element mapping is overall spectrum in Fig. 4S(a). The oxygen (O) was illustrated red dot in

Fig. 4S(b) and Yellow dot for copper (Cu) and blue dot for zinc (Zn) respectively illustrated in Fig.4S(c-d). In Fig. 4S(e) was illustrated the quantitative EDS analysis indicates weight percentages of 23.65% for O, 30.44% for Cu, and 45.92% for Zn, corresponding to atomic percentages of 55.58%, 18.01%, and 26.41%, respectively. The high oxygen content is consistent with the formation of metal oxides, while the relative proportions of Cu and Zn reflect their successful incorporation into the composite matrix. No additional impurity peaks were observed, verifying the elemental purity of the sample. These results confirm the effective formation of a ZnO-CuO nanocomposite.

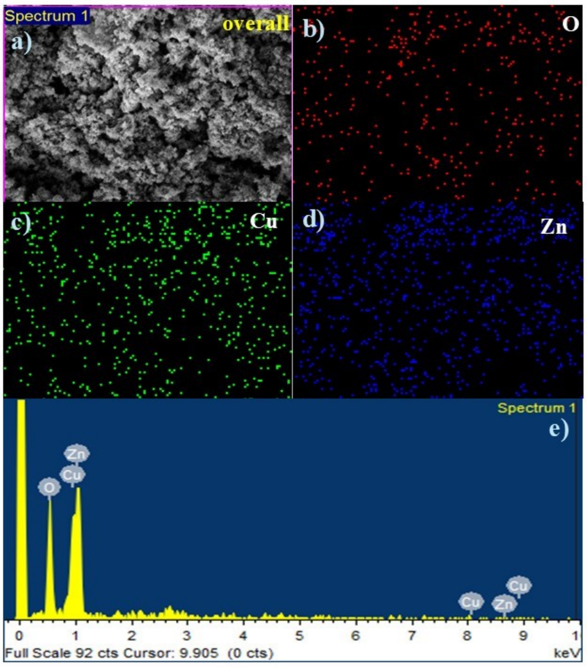

Fig. 4S(a-e) elemental mapping

Photocatalytic degradation performance

The efficiency of ZnO-CuO in photocatalytic degradation was assessed with respect to PRS dye under visible light (Fig.5S). Control experiments (without catalyst) showed negligible degradation, confirming the stability of the pollutants under light exposure alone. Upon addition of ZnO-CuO, PRS exhibited a significant reduction in absorbance intensity at 540 nm, respectively, indicating progressive degradation over time. The normalized concentration (C/C_0) plots (Fig.5S(a–b) further reveal removal efficiencies of 97.91% for 79 min. The initial dark phase ensured equilibrium adsorption. These results demonstrate the high catalytic activity of ZnO-CuO nanocomposite under visible light.

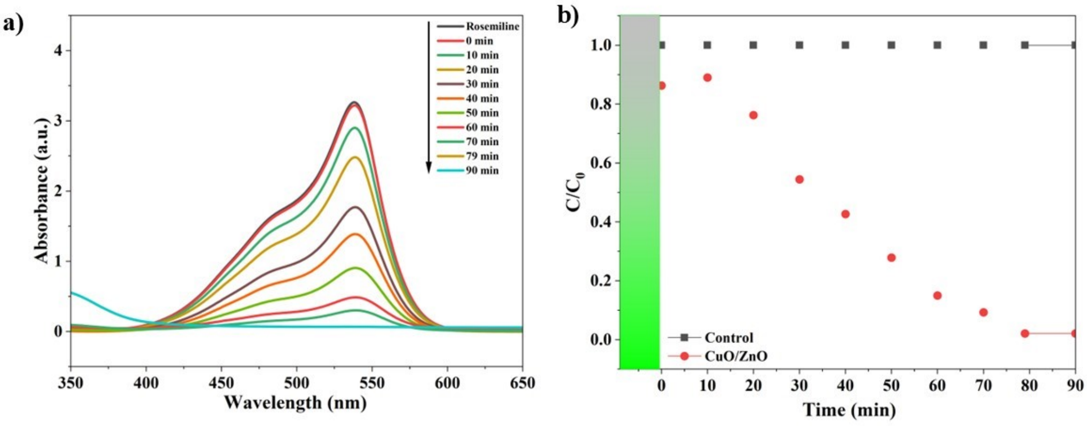

Fig. 5S(a) Absorbance peaks and (b) C/C_0 plots for PRS dye degradation by using ZnO-CuO nanocomposite

Hyperlinks for the Experimental dataset and Synthetic dataset:

Experimental dataset: Data_exp

Synthetic dataset: Data_Syn
